# Supplementary material for: Three-dimensional organization of transzonal projections and other cytoplasmic extensions in the mouse ovarian follicle
Source: Sci Rep. 2019 Feb 4;9:1262. doi: 10.1038/s41598-018-37766-2 (PMC6362238; doi:10.1038/s41598-018-37766-2)
Supplement: Supplementary file 11 — Supplementary materials [file 41598_2018_37766_MOESM11_ESM.pdf]

Supplementary Materials

Three-dimensional organization of transzonal projections and other cytoplasmic extensions in the mouse ovarian follicle

Valentina Baena and Mark Terasaki

Supplementary figures

Supplementary Figure 1. Mitotic cumulus cells have connected and free-ended TZPs. Mitotic cumulus cells were identified by the presence of condensed chromatin and the absence of a nuclear envelope. TZPs derived from this cell were reconstructed from serial sections and then overlaid on the micrograph (orange).

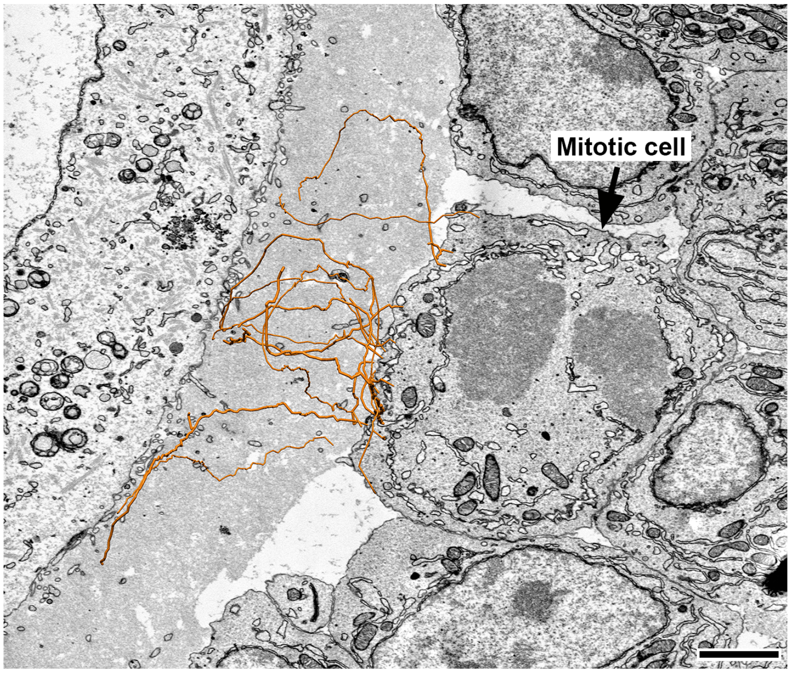

Supplementary Figure 2. TZP branching. A TZP branches into two projections. An asterisk labels the original TZP. Two black arrows label each of the TZP branches. Supplementary Figure 3B shows a reconstruction of TZPs in which two branching points can be seen.

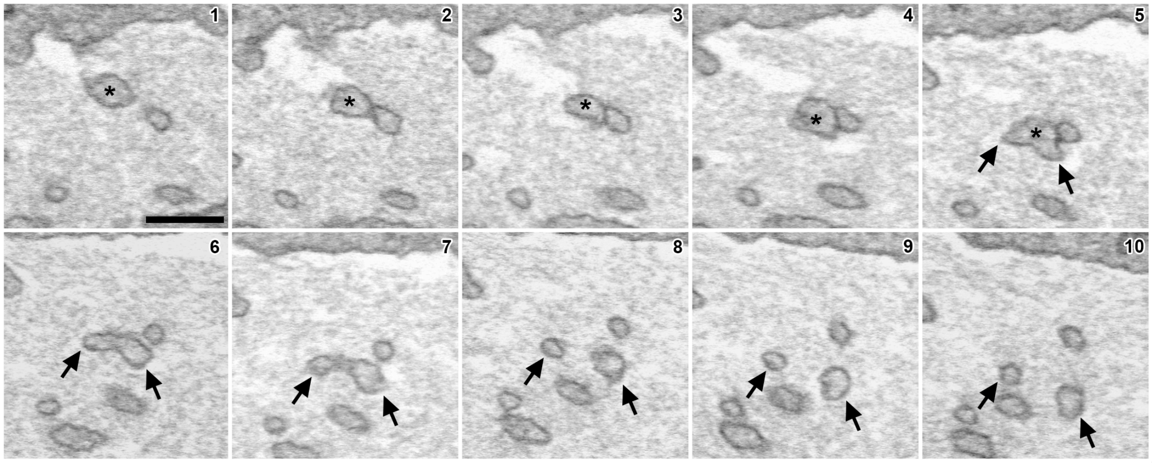

Supplementary Figure 3. TZPs often contact and make gap junctions with each other. (A) Serial section SEM images of two TZPs that contact each other. Scale bar, 250 nm. Video 4 shows three additional examples of contact sites between TZPs. **(B)** Reconstruction of two TZPs derived from different cells that contact each other (black triangle). Asterisks represent branching points on the TZPs (see Supplementary Figure 2). Reconstruction is 4.2 x 4.2 x 5.7 µm (x, y, z), spanning through 126 serial sections (each, 45 nm-thick). Yellow represents the oocyte membrane at the TZP-oocyte junction. **(C)** TEM image of a contact site in the zona pellucida showing a gap junction (high-magnification insert). CC, cumulus cell. ZP, zona pellucida. Scale bar, 500 nm.

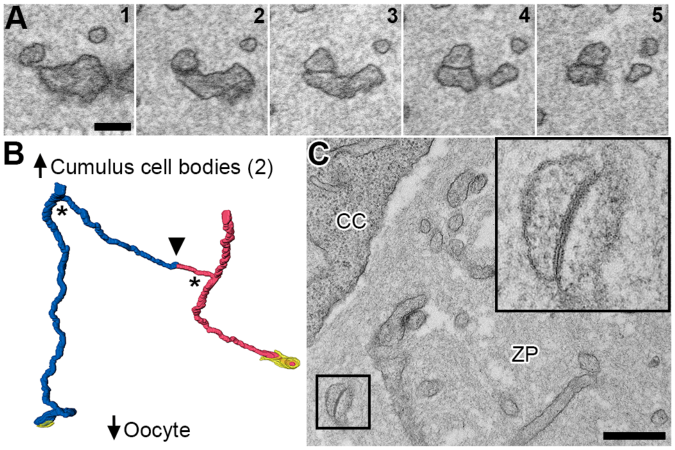

Supplementary Figure 4. TZPs make invaginated junctions with cumulus cell bodies. Black arrow labels a TZP that invaginates the cell body of a cumulus cell. Based on comparison with our previous study, this is most likely an invaginated gap junction (Norris et al., 2017).

Figure legends for supplementary videos

Video 1. Projections can be derived from cumulus cells not directly adjacent to the zona pellucida. 202 serial section SEM images highlighting a cumulus cell that is displaced from the zona pellucida. Connected and free-ended TZPs originate from the cell process at the edge of the zona pellucida. Additionally, several cytoplasmic projections originate from the elongated shaft of the cell and travel in many directions. All cytoplasmic projections originating from this cell body are labeled in green. Same cell shown in Figure 4D (green).

Video 2. Reconstruction of 5 cumulus cell bodies and every cytoplasmic projection derived from them. Reconstruction is 28.4 x 24.6 x 18.2 µm (x, y, z), encompassing 405 serial sections (each, 45 nm-thick). The oocyte and the zona pellucida are located at the bottom. Same reconstruction as Figure 4B.

Video 3. A connected TZP makes a long looping junction with the oocyte surface. Serial section SEM images through the zona pellucida of an antral follicle. Cumulus cell bodies are shown on top, and the oocyte surface is shown on bottom. Asterisk labels a connected TZP that makes a long junction with the oocyte surface and then loops back into the zona pellucida.

Video 4. TZPs make contact sites with each other. Serial electron micrographs showing three examples of contact sites between TZPs. Each contact site is labeled with a differently colored arrow. Most of these contacts were found to be gap junctions by TEM (see Supplementary Figure 3C).

Video 5. Oocyte microvilli closely associate with connected TZPs (serial sections). Red arrow labels a TZP that eventually connects to the oocyte surface. Yellow arrow labels a long microvillus from the oocyte that associates with the TZP for a long distance. The TZP gets surrounded by more microvilli (intermittent yellow arrows) as it gets closer to the oocyte surface. A reconstruction of these structures can be seen in Figure 3F.

Video 6. Inner mural granulosa cells send projections in many directions (serial sections). 240 serial section SEM images of inner mural granulosa cells. Four cells (same as those in Figure 5) are labeled with different colors. Cytoplasmic projections derived from each cell are labeled with the same color as their respective cell body. Several long projections that extend for several cell diameters can be seen originating from every cell. Notice multiple projections that originate from the blue cell and invaginate extensively into a neighboring cell located to its upper right. A rotating reconstruction of these cells can be seen in video 7.

Video 7. Inner mural granulosa cells send projections in many directions (reconstruction). Reconstruction of 4 inner mural granulosa cell bodies and every cytoplasmic projection derived from them. Reconstruction is 21.2 x 16.4 x 20.8 µm (x, y, z), encompassing 462 serial sections (each, 45 nm-thick). Same cells shown in Figure 5 and Video 6.

Video 8. Outer mural granulosa cells send projections in many directions (serial sections). 267 serial section SEM images of outer mural granulosa cells. Four cells (same as those in Figure 6) are labeled with different colors. Cytoplasmic projections derived from each cell are labeled with the same color as their respective cell body. The basal lamina is shown at the top and cell processes from theca and endothelial cells are seen on the opposite side of it (see Figure 6A). Notice that the yellow and light blue cells connect to the basal lamina through a long thick cytoplasmic process. Thin cytoplasmic projections can be seen originating from the cell bodies and the thick cytoplasmic process. Numerous invaginating projections originate from the yellow cell towards a neighboring cell to its right. A rotating reconstruction of these cells can be seen in video 9.

Video 9. Outer mural granulosa cells send projections in many directions (reconstruction). Reconstruction of 4 outer mural granulosa cell bodies and every cytoplasmic projection derived from them. Basal lamina is located at the top. Notice that the further away the cell body is from the basal lamina, the more projections it possesses. Reconstruction is 16.4 x 14.7 x 22.4 µm (x, y, z), encompassing 497 serial sections (each, 45 nm-thick). Same cells shown in Figure 6 and Video 8.

Video 10. Non-TZP cytoplasmic projections often invaginate into neighboring cells. Red arrow labels a cytoplasmic projection derived from a cumulus cell, which invaginates into a neighboring cumulus cell. Notice the space between the invaginated projection and the plasma membrane of the cell into which it invaginates. We have not detected fused membranes at the invaginations. This type of ending is seen in 24% of all non-TZP cytoplasmic projections of every cell type.
